# Phagocytosis influences the intracellular survival of *Mycobacterium smegmatis* via the endoplasmic reticulum stress response

**DOI:** 10.1186/s13578-018-0250-2

**Published:** 2018-09-29

**Authors:** Seon-Hwa Kim, Soo-Na Cho, Yun-Ji Lim, Ji-Ae Choi, Junghwan Lee, Dam Go, Chang-Hwa Song

**Affiliations:** 1 Department of Medical Science, College of Medicine, Chungnam National University, 266 Munhwa-ro, Jung-gu, Daejeon, 35015 South Korea; 2 Research Institute for Medical Sciences, College of Medicine, Chungnam National University, 266 Munhwa-ro, Jung-gu, Daejeon, 35015 South Korea; 3 Department of Microbiology, College of Medicine, Chungnam National University, 266 Munhwa-ro, Jung-gu, Daejeon, 35015 Republic of Korea

**Keywords:** ER stress response, Apoptosis, Phagocytosis, Reactive oxygen species

## Abstract

**Background:**

*Mycobacterium smegmatis*, a rapidly growing non-tuberculosis mycobacterium, is a good model for studying the pathogenesis of tuberculosis because of its genetic similarity to *Mycobacterium tuberculosis* (Mtb). Macrophages remove mycobacteria during an infection. Macrophage apoptosis is a host defense mechanism against intracellular bacteria. We have reported that endoplasmic reticulum (ER) stress is an important host defense mechanism against Mtb infection.

**Results:**

In this study, we found that *M. smegmatis* induced strong ER stress. *M. smegmatis*-induced reactive oxygen species (ROS) play a critical role in the induction of ER stress-mediated apoptosis. Pretreatment with an ROS scavenger suppressed *M. smegmatis*-induced ER stress. Elimination of ROS decreased the ER stress response and significantly increased the intracellular survival of *M. smegmatis*. Interestingly, inhibition of phagocytosis significantly decreased ROS synthesis, ER stress response induction, and cytokine production.

**Conclusions:**

Phagocytosis of *M. smegmatis* induces ROS production, leading to production of proinflammatory cytokines. Phagocytosis-induced ROS is associated with the *M. smegmatis*-mediated ER stress response in macrophages. Therefore, phagocytosis plays a critical role in the induction of ER stress-mediated apoptosis during mycobacterial infection.

## Background

Nontuberculous mycobacteria (NTM) are defined as mycobacteria other than *Mycobacterium tuberculosis* (Mtb), are mycobacteria which do not cause tuberculosis or leprosy [1]. NTM can cause lung, lymphatic, skin/soft tissue, and disseminated disease [1]. Human infection with *Mycobacterium smegmatis* is very rare. *M. smegmatis* belongs to the group of rapidly growing mycobacteria, together with *Mycobacterium abscessus* and *Mycobacterium fortuitum* [2]. *M. smegmatis* has been suggested to be useful in the study of the mechanism of the adaptive response to hypoxia [3].

Macrophages are responsible for detecting, engulfing, and destroying pathogens and apoptotic cells [4]. Mtb-infected macrophages undergo apoptosis and contribute to host immune defense against mycobacterial infection [5, 6]. Apoptosis of *M. smegmatis*-infected macrophages is induced by activation of caspase-3 and tumor necrosis factor-alpha (TNF-α), and regulation of apoptosis is important for the control of *M. smegmatis* in the host [7]. Phosphor-myo-inositol-lipoarabinomannan (LAM) and uncapped (Ara-) LAM have been isolated from the cell walls of *M. smegmatis* and *M. fortuitum*, and these compounds induce apoptosis [7]. Macrophages are also important in the innate immune system, and are involved in pathogen recognition and phagocytosis, as well as the production of cytokines [4, 8]. Phagocytosis is a major mechanism for the removal of pathogens and cell debris, and it is performed by neutrophils, macrophages, dendritic cells, and B lymphocytes [9]. Surface receptors, such as the Fcɣ and complement receptors, are important for phagocytosis by macrophages [10].

Reactive oxygen species (ROS) are associated with inflammatory cytokine production and microbial sterilization in macrophages [11]. Phagocytes generate ROS through the activities of nicotinamide adenine dinucleotide phosphate oxidase (NOX)-family proteins [12]. Activation of NOX2 generates ROS in microorganism-containing phagosomes [12]. ROS production is closely associated with the endoplasmic reticulum (ER) stress response [13].

The ER plays important roles in protein folding, calcium storage, and the transportation of synthesized proteins in vesicles to the Golgi apparatus [14, 15]. ER stress is induced by accumulation of unfolded or misfolded proteins in the ER [16, 17], and chaperone proteins maintain ER homeostasis [17]. ER stress induces an intracellular signaling pathway known as the unfolded protein response (UPR) [18–20]. ER stress-mediated apoptosis plays an important role in the pathogenesis of tuberculosis [18, 19]. ER stress is induced by the 38-kDa antigen and heparin-binding hemagglutinin antigen in macrophages infected with Mtb [6, 21–23]. However, the interplay between ER stress and apoptosis in *M. smegmatis*-infected macrophages has not been studied.

In this study, we found that phagocytosis is important for induction of the ROS-mediated ER stress response in *M. smegmatis*-infected macrophages.

## Results

### *M. smegmatis* induces ER stress-mediated apoptosis of macrophages

**Fig. 1:**
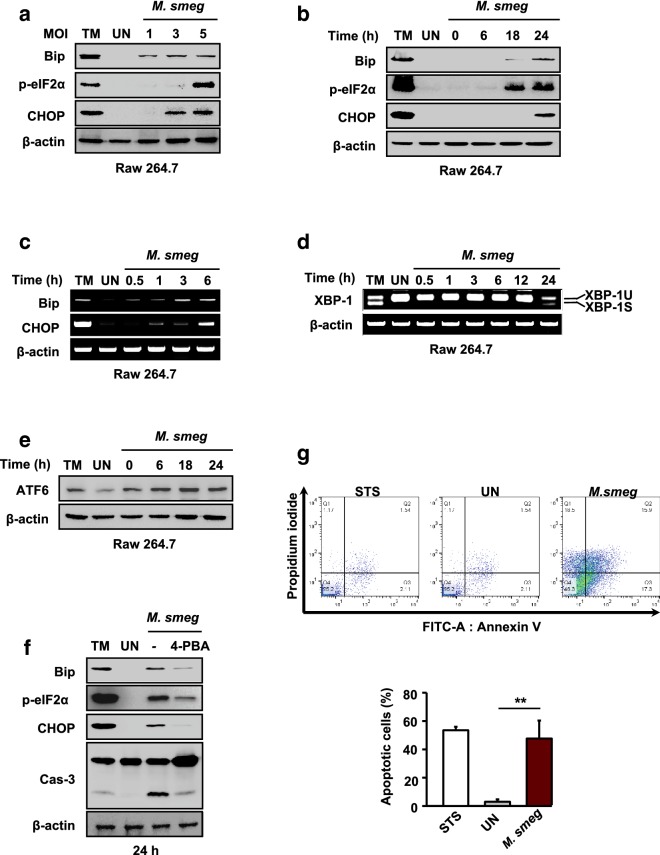
*M. smegmatis* induces strong ER stress-mediated apoptosis in macrophages. **a** RAW 264.7 cells were infected with *M. smegmatis* at multiplicities of infection (MOIs) of 1, 3, and 5 for 24 h. **b** RAW 264.7 cells were infected with *M. smegmatis* (MOI = 5) for the indicated times, and the levels of ER stress molecules were analyzed by western blotting using specific antibodies. **c**, **d** RAW 264.7 cells were infected with *M. smegmatis* at an MOI of 5 and incubated for the indicated times. The mRNA levels of ER stress molecules were determined by RT-PCR. **e** RAW 264.7 cells were infected with *M. smegmatis* (MOI = 5) and incubated for 0–24 h. Induction of ATF6 was analyzed by western blotting using a specific antibody. **f** RAW 264.7 cells were pretreated with 4-PBA (10 mM) for 1 h and infected with *M. smegmatis* (MOI = 5) for 24 h. Western blot analysis was performed using antibodies against ER stress molecules. As a positive control, cells were treated with TM (1 μg/mL) for 6 h. **g** Annexin-V/PI staining was used to evaluate apoptosis of RAW 264.7 cells during *M. smegmatis* infection. Quantification of the flow cytometry results in (**g**) for each culture condition. The percentages of apoptotic cells (sum of early and late apoptotic cells). Data are means ± SDs of three independent experiments. **p < 0.01. *M. smeg*, *Mycobacterium smegmatis;* UN, uninfected; TM, tunicamycin; STS, staurosporine; 4-PBA, 4-phenylbutyric acid

Previous reports suggested that ER stress-mediated apoptosis is associated with mycobacterial killing in macrophages [21, 24]. To investigate whether *M. smegmatis* induces ER stress in macrophages, we analyzed the levels of ER stress sensor molecules in *M. smegmatis*-infected macrophages by western blot analysis. As markers of ER stress response, we monitored the production of CCAAT-enhancer-binding homologous protein (CHOP) and binding immunoglobulin protein (Bip), phosphorylation of eukaryotic translation initiation factor 2α (eIF2α), and spliced X-box binding protein-1 (XBP-1) mRNA. The production of CHOP and Bip, and eIF2α, was increased in macrophages in multiplicity of infection (MOI)- and time-dependent manners (Fig. 1a, b). In order to study the correlation of gene and protein expression with the ER stress markers, we measured the mRNA levels of CHOP and Bip in RAW 264.7 cells after *M. smegmatis* infection. The mRNA levels of CHOP and Bip increased in a time-dependent manner (Fig. 1c). Spliced X-box binding protein-1 (XBP-1) mRNA was found in RAW 264.7 cells at 24 h after *M. smegmatis* infection (Fig. 1d). The expression of activating transcription factor 6 (ATF6) was also increased at 24 h after *M. smegmatis* infection in RAW 264.7 cells (Fig. 1e). The results showed in Fig. 1a–e suggest that ER stress in RAW 264.7 cells is significantly induced by *M. smegmatis* infection.

Next, we examined whether ER stress is involved in *M. smegmatis*-mediated apoptosis. RAW 264.7 cells were pretreated with 4-phenylbutyrate (4-PBA; a chemical chaperone) for 1 h before *M. smegmatis* infection, and caspase-3 activity was evaluated. As expected, the *M. smegmatis*-induced caspase-3 activation and production of CHOP and Bip in macrophages were reduced by 4-PBA pretreatment (Fig. 1f). These results indicate that ER stress is important for the induction of apoptosis in macrophages infected with *M. smegmatis*.

ER stress-mediated apoptosis is significantly increased in macrophages infected with Mtb [21]. We thus investigated whether infection with *M. smegmatis* induces apoptosis. RAW 264.7 cells were infected with *M. smegmatis* for 24 h and subjected to Annexin-V/propidium iodide (PI) staining. Apoptosis was strongly induced in *M. smegmatis*-infected macrophages (Fig. 1g). These data show that *M. smegmatis* induces ER stress-mediated apoptosis of macrophages.

**Fig. 2:**
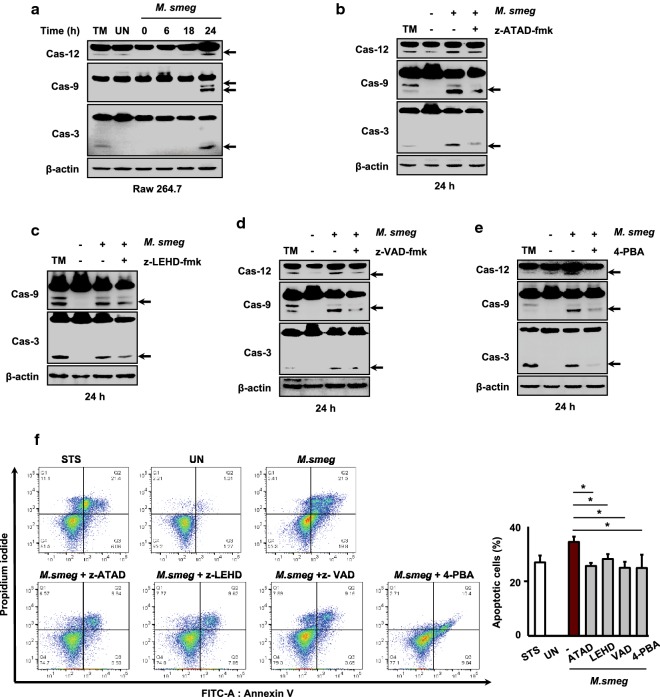
Caspase-12 activation plays a crucial role in *M. smegmatis*-mediated apoptosis. RAW 264.7 cells were infected with *M. smegmatis* (MOI = 5) for 0–24 h. **a** Western blot analysis of caspase-12, -9, and -3 levels. RAW 264.7 cells were pretreated with **b** the caspase-12 inhibitor z-ATAD-fmk (20 μM), **c** the caspase-9 inhibitor z-LEHD-fmk (20 μM), **d** the pan-caspase inhibitor z-VAD-fmk (20 μM), or **e** 4-PBA (10 mM) for 1 h and subsequently infected with *M. smegmatis* (MOI = 5) for 24 h. The activation of caspase-12, -9, and -3 was evaluated by western blotting. **f** RAW 264.7 cells were pretreated with caspase inhibitors and 4-PBA for 1 h and infected with *M. smegmatis* (MOI = 5) for 24 h. Apoptosis was measured by Annexin-V/PI staining. Data are means ± SDs of three independent experiments. *p < 0.05. *M. smeg*, *Mycobacterium smegmatis*; UN, uninfected; TM, tunicamycin; STS, staurosporine; 4-PBA, 4-phenylbutyric acid

Caspase-12 is localized to the cytoplasmic side of the ER membrane [25] and it is translocated from the ER to the cytosol in the presence of ER stress [26]. Caspase-12 directly cleaves caspase-9 and caspase-3, leading to apoptosis [26]. RAW 264.7 cells were infected with *M. smegmatis* and caspase activation was evaluated by western blotting. Activation of caspase-12, -9 and -3 was increased in RAW 264.7 cells infected with *M. smegmatis* at 24 h (Fig. 2a).

To examine which caspase activation is important for the induction of *M. smegmatis*-mediated apoptosis in macrophages, RAW 264.7 cells were pretreated with z-ATAD-fmk (caspase-12 inhibitor), z-LEHD-fmk (caspase-9 inhibitor), and z-VAD-fmk (pan-caspase inhibitor) following infection with *M. smegmatis*, and caspase activation was evaluated. The activation of caspase-9 and -3 was strongly decreased by caspase-12 inhibition at 24 h after *M. smegmatis* infection (Fig. 2b). In RAW 264.7 cells pretreated with z-LEHD-fmk (caspase-9 inhibitor), the caspase-9 and -3 levels were slightly decreased at 24 h after *M. smegmatis* infection (Fig. 2c). Pretreatment with z-VAD-fmk reduced the activation of caspase-12, -9, and -3 in *M. smegmatis*-infected macrophages (Fig. 2d). In RAW 264.7 cells pretreated with 4-PBA for 1 h before *M. smegmatis* infection, the activation of caspase-12, -9, and -3 was reduced (Fig. 2e). Next, we assessed the proportion of apoptotic cells by Annexin-V/PI staining in the presence of specific caspase inhibitors and 4-PBA at 24 h after infection with *M. smegmatis*. Pretreatment with each inhibitor and 4-PBA reduced the number of apoptotic cells after *M. smegmatis* infection in macrophages (Fig. 2f). These findings indicate that ER stress plays a key role in caspase-dependent induction of apoptosis in *M. smegmatis*-infected macrophages.

### Roles of nuclear factor kappa B and c-Jun N-terminal kinase in *M. smegmatis*-induced ER stress

**Fig. 3:**
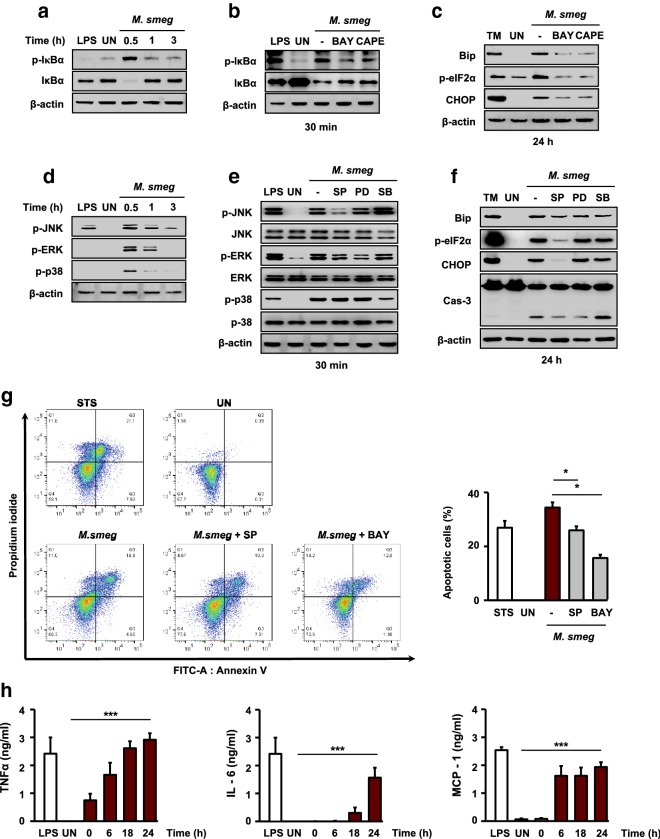
NF-κB and JNK play key roles in *M. smegmatis*-induced ER stress. RAW 264.7 cells were infected with *M. smegmatis* (MOI = 5) for the indicated times. **a** Total cell lysates were analyzed by western blot detecting p-IκBα. **b** RAW 264.7 cells were pretreated with BAY (10 μM) or CAPE (10 μM) for 1 h and infected with *M. smegmatis*. Phosphorylation of IκBα was analyzed by western blotting. **c** Western blot analysis was performed using specific antibodies against Bip, p-eIF2α, and CHOP. **d** MAPK (p-JNK, p-ERK, and p-p38) was analyzed by western blotting. **e** RAW 264.7 cells were pretreated with SP (20 μM), PD (50 μM), and SB (10 μM) for 1 h and infected with *M. smegmatis*. Western blot analysis was performed using specific antibodies against p-JNK, p-ERK, p-p38, **f** Bip, p-eIF2α, CHOP, and caspase-3. **g** RAW 264.7 cells were pretreated with SP (20 μM) or BAY (10 μM) and subsequently infected with *M. smegmatis* for 24 h. Apoptosis was assayed by Annexin-V/PI staining. **h** Inflammatory cytokine levels were determined by ELISA at the indicated time points. LPS (500 ng/mL, 24 h) was used as the positive control. Data are means ± SDs of three independent experiments. *p < 0.05, ***p < 0.001. *M. smeg*, *Mycobacterium smegmatis*; UN, uninfected; TM, tunicamycin; STS, staurosporine; LPS, lipopolysaccharide; SP, SP600125; PD, PD98059; SB, SB203580; BAY, Bay11–7082; CAPE, caffeic acid phenethyl ester

Under stress conditions, the UPR induces nuclear factor kappa B (NF-κB) activation [27]. To examine the relationship between the NF-κB pathway and *M. smegmatis*-mediated ER stress, we first evaluated the activation of NF-κB and mitogen-activated protein kinase (MAPK). RAW 264.7 cells were infected with *M. smegmatis* for 0–3 h. Phosphorylation of inhibitory proteins of κB family (IκB) was strongly induced at 30 min after *M. smegmatis* infection (Fig. 3a), and thereafter decreased gradually. RAW 264.7 cells were pretreated with NF-κB inhibitors (Bay11-7082 and CAPE) for 1 h before *M. smegmatis* infection (Fig. 3b). Inhibition of NF-κB considerably reduced the levels of ER stress sensor molecules (Fig. 3c).

MAPK activation was observed at 30 min after *M. smegmatis* infection (Fig. 3d). *M. smegmatis*-induced MAPK activation was reduced by each MAPK pathway inhibitor (Fig. 3e). Interestingly, pretreatment with SP600125 [c-Jun N-terminal kinase (JNK) inhibitor] substantially reduced the levels of ER stress molecules in *M. smegmatis* infected-macrophages (Fig. 3f). Inhibition of the endoplasmic reticulum kinase (ERK) or p38 pathway did not affect the induction of *M. smegmatis*-mediated ER stress (Fig. 3f). Moreover, apoptosis was decreased in *M. smegmatis*-infected macrophages by pretreatment with a JNK inhibitor and an NF-κB inhibitor (Fig. 3g). The levels of proinflammatory cytokines, such as IL-6, TNF-α, and MCP-1, increased in a time-dependent manner during *M. smegmatis* infection (Fig. 3h). Our data indicate that the JNK pathway is important for ER stress-mediated apoptosis of *M. smegmatis*-infected macrophages.

### ROS-mediated ER stress modulates intracellular survival of *M. smegmatis* in macrophages

**Fig. 4:**
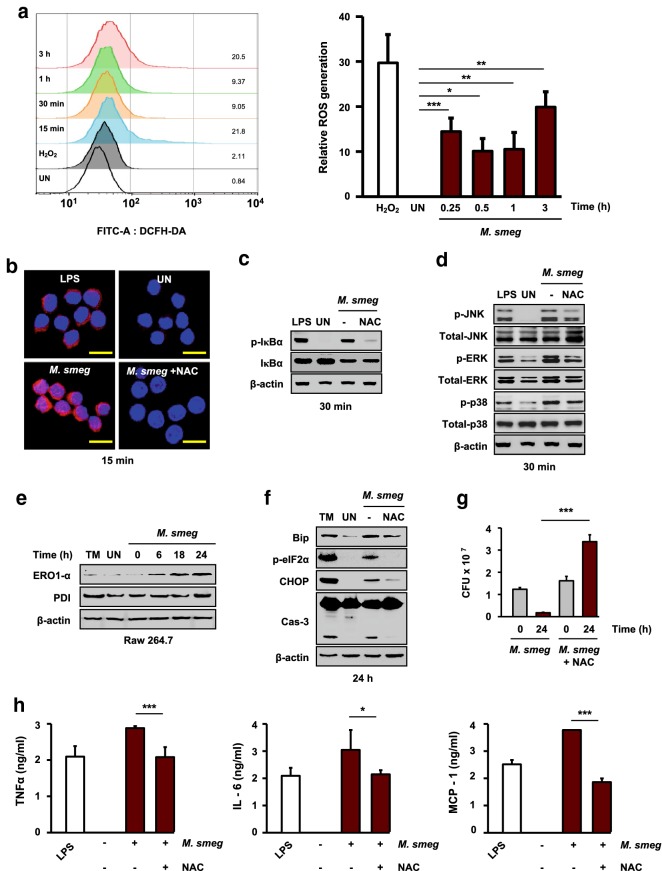
Phagocytosis and ROS induce the production of proinflammatory cytokines in *M. smegmatis*-infected macrophages. RAW 264.7 cells were infected with *M. smegmatis* (MOI = 5) for the indicated times. **a** Intracellular ROS levels were determined by DCFH-DA (5 μM) staining and FACS analysis. H_2_O_2_ (1 mM, 1 h) was used as the positive control. **b** RAW 264.7 cells were pretreated with NAC (30 mM) for 1 h before *M. smegmatis* infection. Intracellular ROS levels were determined by DHE (2 μM) staining (scale bar = 10 μm). **c**, **d** NAC-pretreated RAW 264.7 cells were infected with *M.* *smegmatis* for 30 min. NF-κB and MAPK activation was measured by western blot analysis. **e** Western blot analysis was performed using specific antibodies against ERO1-α and PDI. NAC-pretreated RAW 264.7 cells were infected with *M. smegmatis* (MOI = 5) for 24 h. **f** Western blot analysis was performed using specific antibodies against Bip, p-eIF2α, CHOP, and caspase-3. **g** Numbers of intracellular *M. smegmatis* in RAW 264.7 cells were determined by plate counting. **h** Inflammatory cytokine levels were determined by ELISA at 24 h. LPS (500 ng/mL, 24 h) was used as the positive control. Data are means ± SDs of at least three independent experiments. *p < 0.05, **p < 0.01, ***p < 0.001. *M. smeg*, *Mycobacterium smegmatis*; UN, uninfected; TM, tunicamycin; LPS, lipopolysaccharide; DCFH-DA, dichlorofluorescein diacetate; DHE, dihydroethidium; NAC, *N*-acetyl-cysteine

Mtb-generated ROS induce ER stress-mediated apoptosis [28]. ROS lead to accumulation of misfolded proteins in the ER, which drives the ER stress response [29]. Therefore, we investigated the relationship between ROS and ER stress in macrophages infected with *M. smegmatis* by measuring intracellular ROS levels in RAW 264.7 cells. The ROS level was significantly increased in RAW 264.7 cells infected with *M. smegmatis* (Fig. 4a). *N*-acetyl-cysteine (NAC; ROS scavenger) pretreatment resulted in a reduced ROS level in *M. smegmatis*-infected macrophages at 15 min (Fig. 4b). NAC pretreatment also reduced the levels of IκBα and MAPK phosphorylation in *M. smegmatis*-infected macrophages (Fig. 4c, d). We next assayed the expression of two ROS generation-related enzymes, protein disulfide isomerase (PDI) and endoplasmic reticulum oxidoreductase 1 alpha (ERO1α), in *M. smegmatis*-infected macrophages by western blotting. The ERO1α and PDI levels were increased in RAW 264.7 cells infected with *M. smegmatis* at 24 h (Fig. 4e). Moreover, NAC pretreatment reduced the levels of ER stress molecules in *M. smegmatis*-infected macrophages (Fig. 4f). Furthermore, intracellular survival of *M.* *smegmatis* was significantly increased by NAC treatment at 24 h (Fig. 4g). To examine the relationship between proinflammatory cytokine production and ROS in *M.* *smegmatis*-infected macrophages, RAW 264.7 cells were pretreated with NAC for 1 h before *M. smegmatis* infection. Inhibition of intracellular ROS production resulted in reduced levels of proinflammatory cytokines in *M. smegmatis*-infected macrophages (Fig. 4h). ROS generation is important for the production of proinflammatory cytokines during *M. smegmatis* infection. These results suggest that *M. smegmatis*-induced ROS activate the ER stress response through the MAPK and NF-κB pathways, leading to suppression of the intracellular survival of *M. smegmatis* in macrophages.

### Phagocytosis is important for the induction of ER stress during *M. smegmatis* infection

**Fig. 5:**
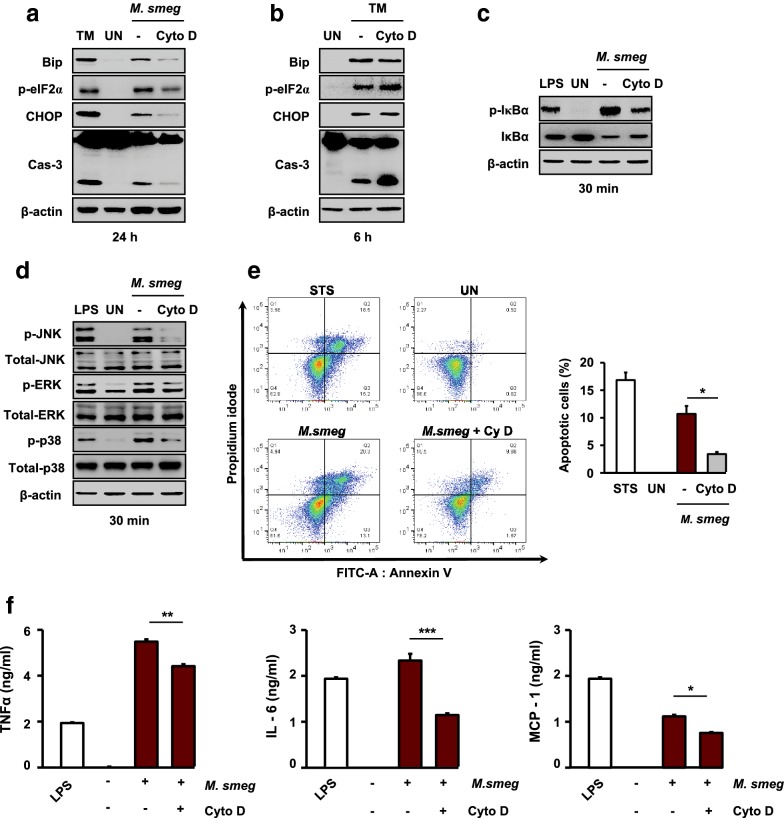
*M. smegmatis*-induced ROS activate the ER stress response via the MAPK and NF-κB pathways. **a**, **b** RAW 264.7 cells were pretreated with the phagocytosis inhibitor Cyto D (20 μM) for 1 h and infected with *M. smegmatis* (MOI = 5) for 24 h or stimulated with TM (1 μg/mL) for 6 h. Western blot analysis was performed using specific antibodies against Bip, p-eIF2α, CHOP, and caspase-3. **c**, **d** RAW 264.7 cells were pretreated with Cyto D (20 μM) for 1 h and infected with *M. smegmatis* for 30 min. NF-κB and MAPK activation was measured by western blot analysis. **e** RAW 264.7 cells were pretreated with the phagocytosis inhibitor Cyto D (20 μM) for 1 h and infected with *M. smegmatis* (MOI = 5) for 24 h. Apoptosis was measured by Annexin-V/PI staining and FACS analysis. STS was used as the positive control. **f** RAW 264.7 cells were pretreated with Cyto D (20 μM) or NAC (30 mM) for 1 h and infected with *M. smegmatis* (MOI = 5) for 24 h. Inflammatory cytokine levels were determined by ELISA at 24 h. LPS (500 ng/mL, 24 h) was used as the positive control. Data are means ± SDs of three independent experiments. *p < 0.05, **p < 0.01, ***p < 0.001. *M. smeg*, *Mycobacterium smegmatis*; UN, uninfected; TM, tunicamycin; STS, staurosporine; LPS, lipopolysaccharide; Cyto D, cytochalasin D

Phagocytosis of pathogens by macrophages is a key innate immune effector mechanism [30]. Therefore, phagocytosis may be related to ER stress during mycobacterial infection. To examine the relationship between phagocytosis and the ER stress response, RAW 264.7 cells were pretreated with cytochalasin D (phagocytosis inhibitor) for 1 h before *M. smegmatis* infection. The expression of Bip, CHOP, and caspase-3 was significantly reduced by cytochalasin D pretreatment in macrophages infected with *M. smegmatis* (Fig. 5a). We next examined whether *M. smegmatis*-induced ER stress is dependent on phagocytosis of *M. smegmatis*. RAW 264.7 cells were pretreated with cytochalasin D and then treated with tunicamycin (TM) for 6 h. Interestingly, cytochalasin D pretreatment before TM stimulation did not inhibit the TM-mediated ER stress response (Fig. 5b). Moreover, activation of NF-κB and MAPK was suppressed by cytochalasin D pretreatment in *M. smegmatis*-infected macrophages (Fig. 5c, d). Cytochalasin D pretreatment also reduced the percentage of apoptotic macrophages infected with *M. smegmatis* (Fig. 5e). Next, we examined the relationship between proinflammatory cytokines and phagocytosis during *M. smegmatis* infection. Cytochalasin D pretreatment resulted in reduced IL-6, TNF-α, and MCP-1 levels in RAW 264.7 cells infected with *M. smegmatis* at 24 h (Fig. 5f).

These data suggest that phagocytosis plays a key role in the induction of ER stress during *M. smegmatis* infection.

## Discussion

Apoptosis is important for host defense against mycobacterial infection [31]. In this study, we found that avirulent *M. smegmatis* induces ER stress-mediated apoptosis in macrophages. Avirulent mycobacteria induce stronger ER stress responses than do virulent mycobacteria [5, 24]. Our previous report suggested that virulent mycobacteria modulate macrophage polarization to control ER stress [5]. Toll-like receptor (TLR)2- and TLR4-mediated signaling typically plays a pivotal role in proinflammatory cytokine production or apoptosis of macrophages induced by Mtb [32]. The question remained of whether ER stress is induced in the presence of functional TLR signaling. Because TLR signaling is not directly involved in phagocytosis of mycobacteria, we postulated that ER stress would occur even when phagocytosis was inhibited. Unexpectedly, cytochalasin D pretreatment reduced *M. smegmatis*-induced ER stress in macrophages. In addition, *M. smegmatis*-induced production of proinflammatory cytokines was abolished by pretreatment with cytochalasin D or NAC in macrophages. These results suggest that direct phagocytosis of *M. smegmatis* plays a crucial role in overproduction of proinflammatory cytokines as well as TLR signaling.

Phagocytosis is an important mechanism for the removal of pathogens and cell debris. Phagocytosis of invading pathogens by macrophages is the first line of defense and initiates the cellular immune response [9]. Phagocytosed pathogens, such as Mtb, upregulate ROS production, leading to activation of MAPK and NF-κB [33]. Here, we showed that ROS production in *M. smegmatis*-infected macrophages was reduced by cytochalasin D pretreatment. Previously, we reported that Mtb-mediated ROS induced accumulation of misfolded proteins in the ER, leading to induction of the ER stress response [6, 24]. These findings indicate that direct phagocytosis of *M. smegmatis* is important for induction of the ROS-mediated ER stress response.

*Mycobacterium smegmatis*-induced ER stress apoptosis was found to be dependent on the JNK pathway. JNK-mediated ER stress activates death-receptor 5, Puma, and Bim via the inositol-requiring enzyme 1 alpha (IRE1α)- TNF receptor-associated factor 2 (TRAF 2)-apoptosis signal-regulating kinase 1 (ASK 1) complex, resulting in apoptosis [34]. Therefore, we suggest that ER stress plays a key role in the induction of apoptosis in *M. smegmatis*-infected macrophages, leading to suppression of intracellular survival of the bacilli.

In summary, our results indicate that direct phagocytosis of *M. smegmatis* induces ROS production, which not only leads to the production of proinflammatory cytokines, but also induces ER stress-mediated apoptosis. Therefore, phagocytosis might be an important factor that inhibits intracellular growth of mycobacteria by inducing ER stress-mediated apoptosis in macrophages.

## Methods

### Cell culture

Murine macrophage RAW 264.7 cells were cultured in Dulbecco’s modified Eagle’s medium supplemented with 10% heat-inactivated fetal bovine serum, penicillin (100 IU/mL), and streptomycin (100 mg/mL) at 37 °C with 5% CO_2_. The cells were cultured in six-well polypropylene tissue culture plates in a 5% CO_2_ atmosphere at 37 °C for 24 h before infection to allow them to adhere.

### *M. smegmatis* culture and intracellular survival assay

*Mycobacterium smegmatis* (ATCC 700084/mc^2^ 155) was cultured in Middlebrook 7H9 liquid medium supplemented with 10% oleic acid, albumin, dextrose, and catalase, and 5% glycerol. *M. smegmatis* was harvested by centrifugation (3000 rpm, 30 min), washed, and resuspended in phosphate-buffered saline (PBS) to a concentration of 5 × 10^7^ CFU/mL. The bacterial cells were stored at − 80 °C until used. Heat-killed *M. smegmatis* was prepared by heating of live *M. smegmatis* in PBS at 80 °C for 30 min.

Cells were infected with *M. smegmatis* at an MOI of 5 and incubated for 1 h at 37 °C in a 5% CO_2_ atmosphere. After allowing time for phagocytosis, the cells were washed with PBS to remove extracellular bacteria, and incubated with fresh medium without antibiotics for a further. To assay intracellular survival, *M. smegmatis*-infected cells were lysed in sterile distilled water and disintegrated in a water bath sonicator for 3 min to collect intracellular bacteria. The lysates were plated separately on Middlebrook 7H10 agar plates and incubated for 14–21 days. Colony counts were performed in triplicate.

### Chemicals and antibodies

Chemicals and inhibitors were dissolved in dimethyl sulfoxide and diluted to the desired concentrations directly in the culture medium. RAW 264.7 cells were pretreated with the indicated concentrations of inhibitors for 1 h before *M. smegmatis* infection. TM, z-LEHD-fmk (caspase-9 inhibitor), z-VAD-fmk (pan-caspase inhibitor), SP600125 (p-JNK inhibitor), PD98059 (p-ERK inhibitor), SB203580 (p-p38 inhibitor), BAY 11-7082 (NF-κB inhibitor), and caffeic acid phenethyl ester (NF-κB inhibitor) were purchased from Calbiochem (San Diego, CA, USA). NAC, cytochalasin D, staurosporine, and 4-PBA were purchased from Sigma-Aldrich (St. Louis, MO, USA). Z-ATAD-fmk (caspase-12 inhibitor) was purchased from R&D Systems (Minneapolis, MN, USA). Lipopolysaccharide and H_2_O_2_ were purchased from InvivoGen (San Diego, CA, USA).

Western blotting was performed using antibodies against caspase-12, caspase-9, caspase-3, PDI, ERO1α, p-protein kinase RNA-like ERK, p-eIF2α, CHOP, Bip, p-p38, p-JNK, p-ERK, p-IκBα, IκBα, JNK, ERK, p38 (Cell Signaling, Danvers, MA, USA), and ATF6α (Santa Cruz Biotechnology, Santa Cruz, CA, USA). Goat anti-rabbit IgG (Cell Signaling) and goat anti-mouse IgG (Calbiochem) were used as secondary antibodies. β-actin (Santa Cruz Biotechnology) was used as a loading control.

### Western blot analysis

Whole cells were lysed in radio-immunoprecipitation assay buffer (ELPIS Biotech, Daejeon, Korea) containing a protease inhibitor cocktail. Extracted proteins were separated on 10% or 12% sodium dodecyl sulfate polyacrylamide gel electrophoresis (SDS-PAGE) gels and transferred to polyvinylidene difluoride membranes. The membranes were blocked with Tris-buffered saline, 0.1% Tween 20 (TBS-T) containing 5% skim milk (Santa Cruz Biotechnology) at room temperature for 1 h and then incubated with primary antibodies (1:1000) overnight at 4 °C. After washing with TBS-T, the membranes were incubated with horseradish peroxidase (HRP)-conjugated secondary antibodies (1:2000) for 2 h at room temperature. The bound antibodies were detected using a chemiluminescent HRP substrate (Enhanced chemiluminescence (ECL); Millipore, Billerica, MA, USA). Band intensities were quantitated using Omega Lum C (Aplegen Inc., Pleasanton, CA, USA).

### Apoptosis analysis

Apoptosis was assessed using an Annexin-V-PI staining kit according to the manufacturer’s instructions (BD Pharmingen, San Diego, CA, USA). Cells were analyzed using a fluorescence-activated cell sorting (FACS) Canto II flow cytometer (BD Biosciences, San Jose, CA, USA) and the data were processed using Flow Jo software (Tree Star, Ashland, OR, USA). Staurosporine (500 nM, 6 h) was used as the positive control.

### Measurement of ROS levels

RAW 264.7 cells were harvested using PBS. For ROS staining, the cells were incubated with dichlorofluorescein diacetate (DCFH-DA; 5 μM; Molecular Probes, Eugene, OR, USA) or dihydroethidium (DHE; 2 μM; Molecular Probes) for 30 min in a 5% CO_2_ atmosphere at 37 °C. RAW 264.7 cells were washed three times with PBS and fixed with 4% paraformaldehyde. After being washed with PBS, DCFH-DA-positive cells were analyzed in a FACS Canto II cytometer (BD Biosciences) and the data were processed using Flow Jo software (Tree Star). DHE staining was analyzed using an optical microscope (Olympus BX51, Olympus, Japan). H_2_O_2_ was used as the positive control.

### Reverse transcription polymerase chain reaction

Total RNA was extracted from RAW 264.7 cells infected with *M. smegmatis* using TRIzol™ reagent (Invitrogen, Carlsbad, CA, USA) according to the manufacturer’s instructions. The extracted mRNA was transcribed into cDNA using a reverse transcription kit (ELPIS Biotech). cDNA was amplified using Prime Taq Premix (Genet Bio, Daejeon, Korea) to assess XBP-1 splice, CHOP, and Bip mRNA levels. β-actin was used as the control.

### Enzyme-linked immunosorbent assay

A sandwich enzyme-linked immunosorbent assay (ELISA) kit (BD Biosciences) was used to quantify TNF-α, MCP-1, and IL-6 levels in the culture supernatants of *M. smegmatis*-infected RAW 264.7 cells. Assays were performed following the manufacturer’s instructions. Triplicate samples were analyzed using an ELISA reader and compared with a standard curve.

### Statistical analyses

All experiments were performed in at least triplicate, and representative results are presented. Statistical significance was analyzed using Prism 5 software (GraphPad Software, Inc., San Diego, CA, USA) by one-way analysis of variance. The data are expressed as means ± standard deviations. Statistical significance was set to *p *< 0.05, *p *< 0.01, and *p *< 0.001.
